# Highlight report: Role of PD-L1 in never-smokers

**DOI:** 10.17179/excli2019-1516

**Published:** 2019-06-18

**Authors:** Wiebke Albrecht

**Affiliations:** 1 Leibniz Research Centre for Working Environment and Human Factors at the Technical University of Dortmund (IfADo)

## ⁯⁯

Numerous studies have shown that infiltration of lymphocytes into tumor tissue is associated with better prognosis (Bindea et al., 2013[2]; Lohr et al., 2013[17]; Schmidt et al., 2018[25]; Heimes et al., 2017[12][13]). This prognostic influence has been seen in many tumor types, including colon (Schmidt et al., 2012[24]), breast (Lohr et al., 2013[17]; Schmidt et al., 2008[23]; Godoy et al., 2014[10]) and lung (Botling et al., 2013[3]; Grinberg et al., 2017[11]; Jabs et al., 2017[16]). With the introduction of programmed death ligand 1 (PD-L1) targeting therapy, immune-inhibitory mechanisms have become a major field of research (Garon et al., 2015[9]; Rizvi et al., 2015[22][21]; Herbst et al., 2016[15]; Pardoll, 2012[20]; Creelan, 2014[7]; Aguiar et al., 2017[1]). However, the prognostic role of PD-L1 in non-small cell lung cancer (NSCLC) in patients not treated with PD-1 targeting therapies still is unclear. To gain a better understanding, a study recently published in the Journal of Thoracic Oncology immunohistochemically analyzed tissue microarrays of 705 patients with NSCLC and additionally considered publicly available transcriptomics data of 1724 patients (Edlund et al., 2019[8]). Key findings of this study in patients without PD-1 targeting therapies are: (1) Infiltration of T-, B- and plasma cells is associated with better prognosis, similar to previous studies; (2) This association is strongest in highly proliferative tumors; (3) PD-L1 is not associated with prognosis in the total cohort of NSCLC patients; (4) However, a significant association of PD-L1 positivity with shorter survival was obtained in the never-smokers. This association was validated in independent patients on the RNA level.

This result is of relevance, because it shows that immune modifiers have different roles in NSCLC of smokers and non-smokers. It will be important to consider this difference in therapy studies targeting the PD-1/PD-L1 axis. Predicting prognosis and response to therapy remains a challenging task (Hellwig et al., 2016[14]; Lohr et al., 2015[18]; Weisner et al., 2019[28]) with many modifying factors, e.g. proliferation (Siggelkow et al., 2012[26]), antioxidative status (Cadenas et al., 2010[4], 2014[5], 2019[6]) and metabolism (Marchan et al., 2017[19]; Stewart et al., 2012[27]) playing an important role. The present study of Edlund and colleagues helps to gain an overview which key factors modify the prognostic role of tumor infiltrating lymphocytes. 

## Conflict of interest

The author declares no conflict of interest.
